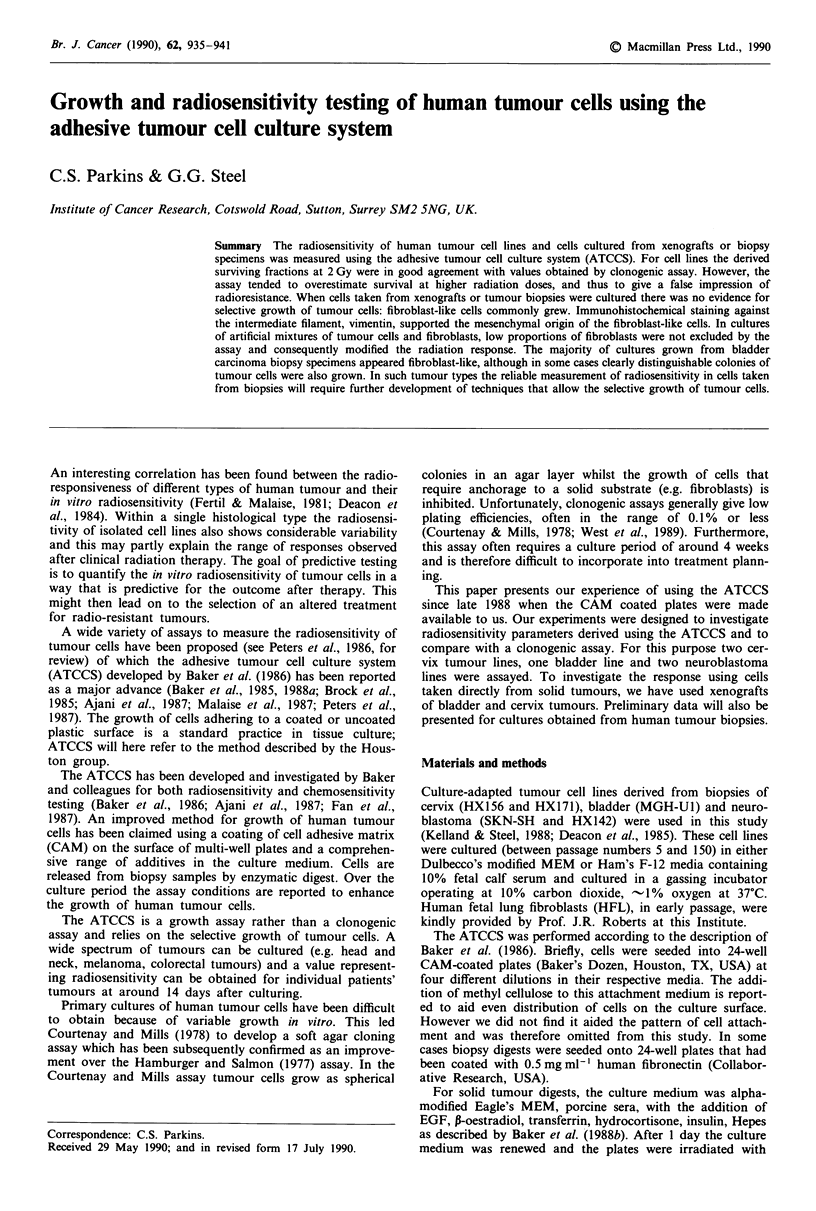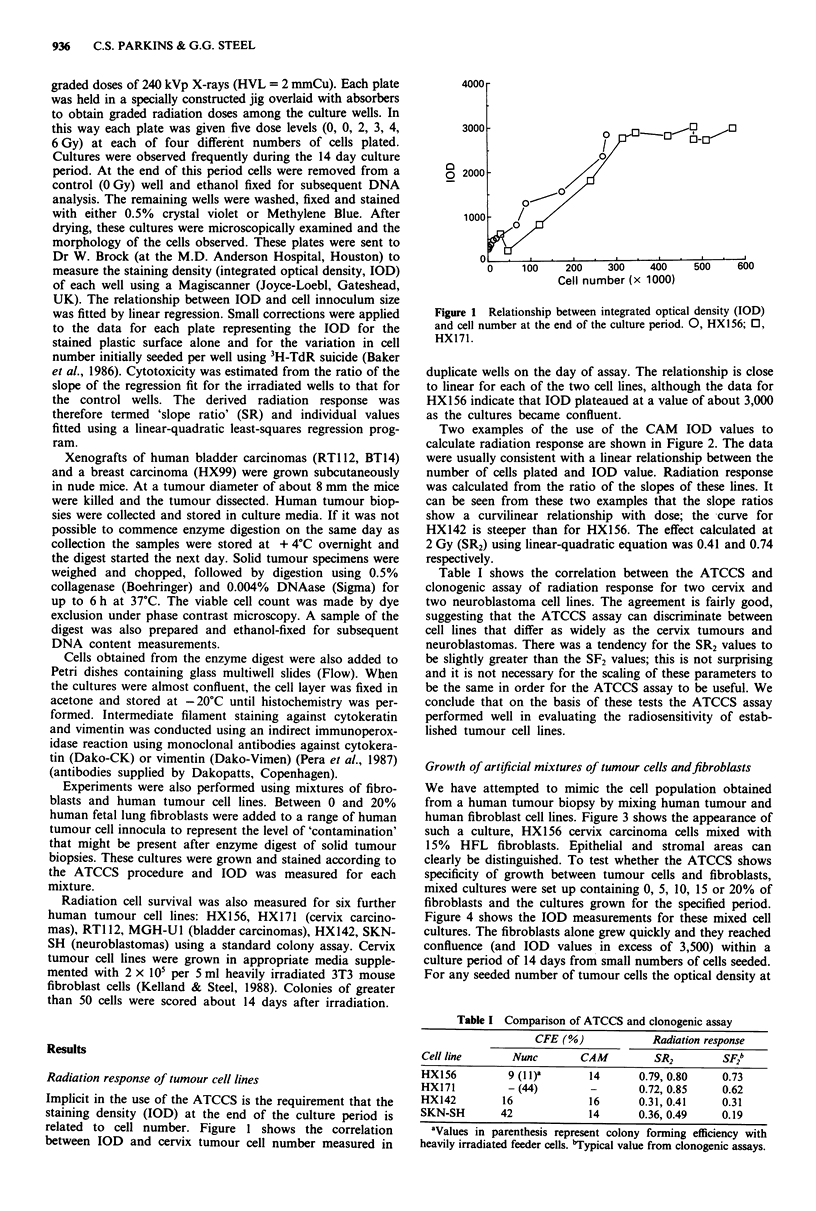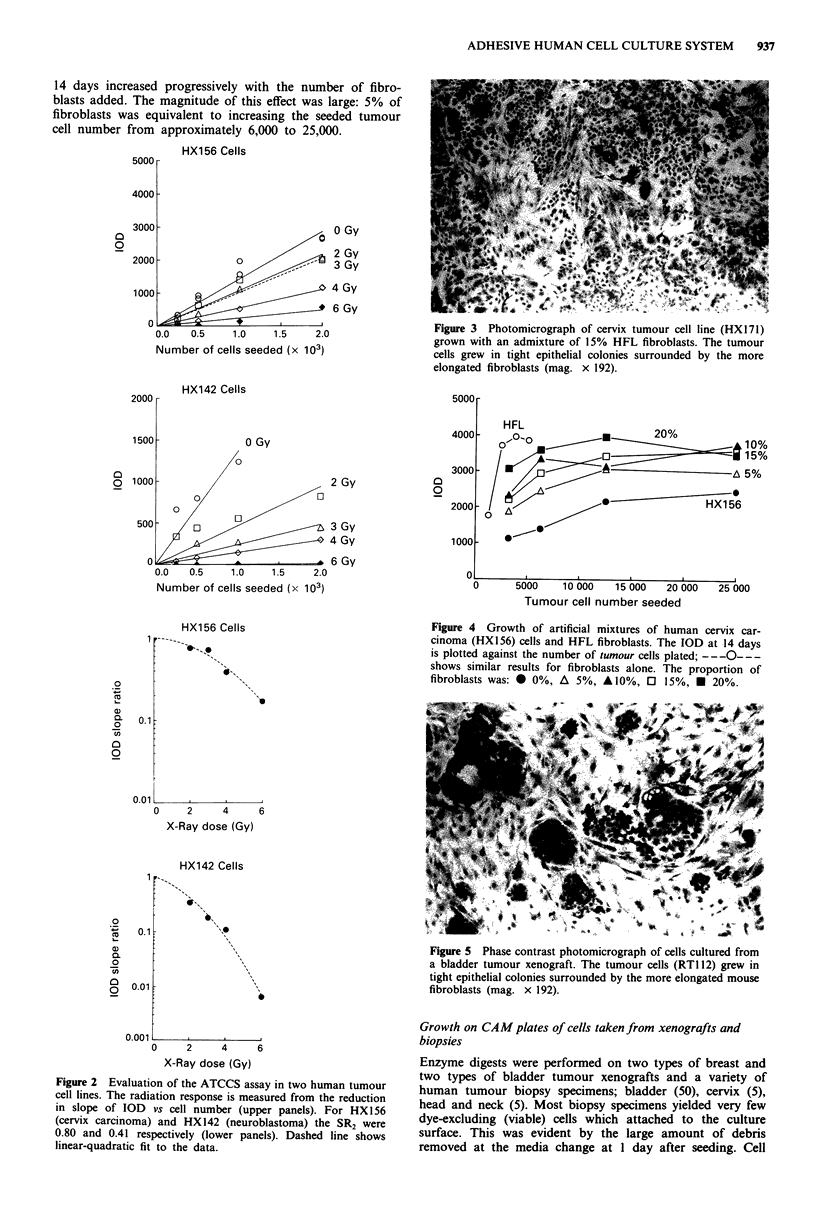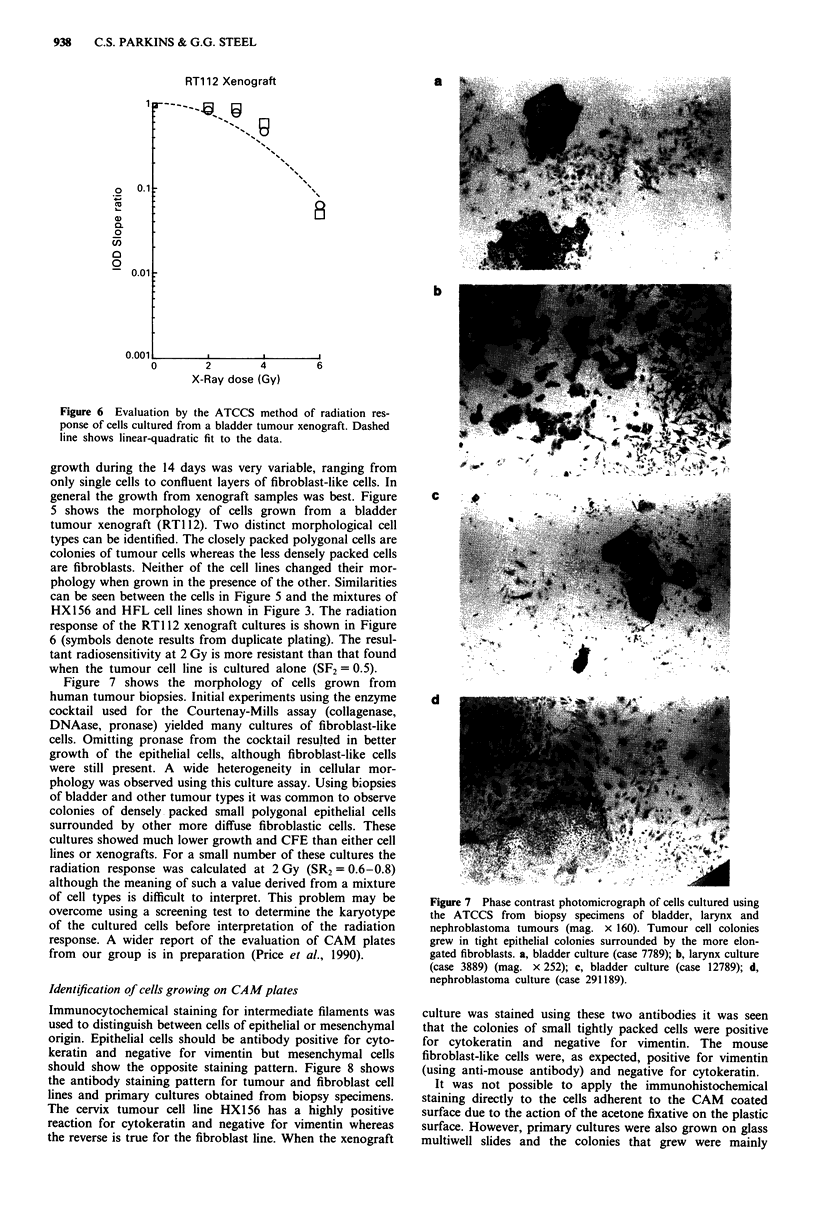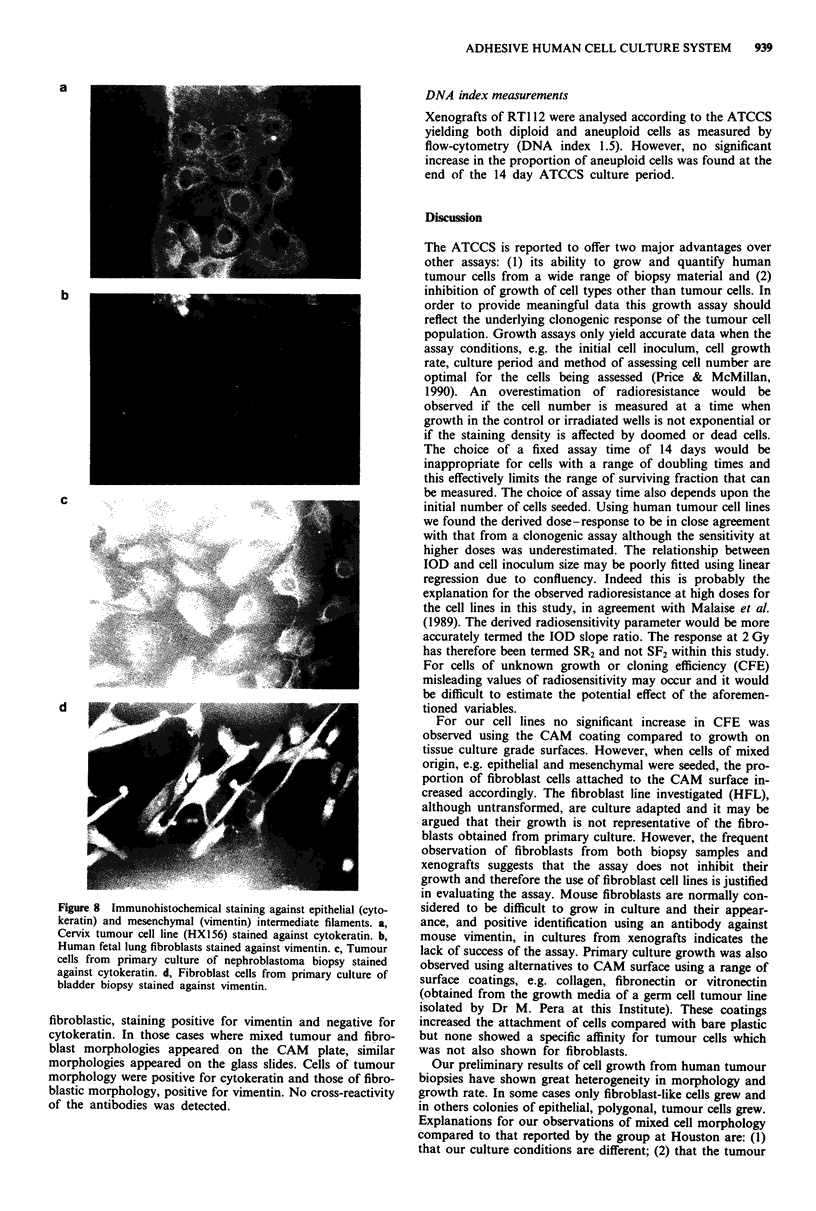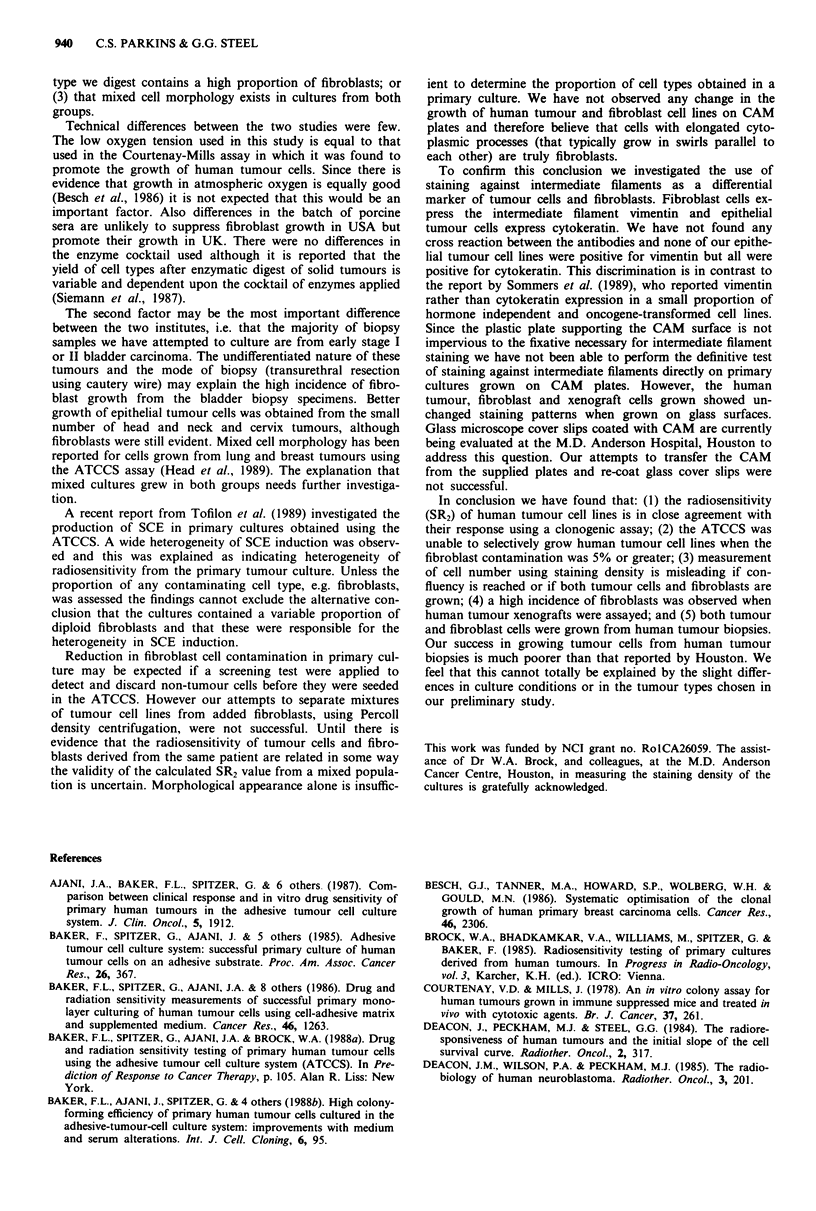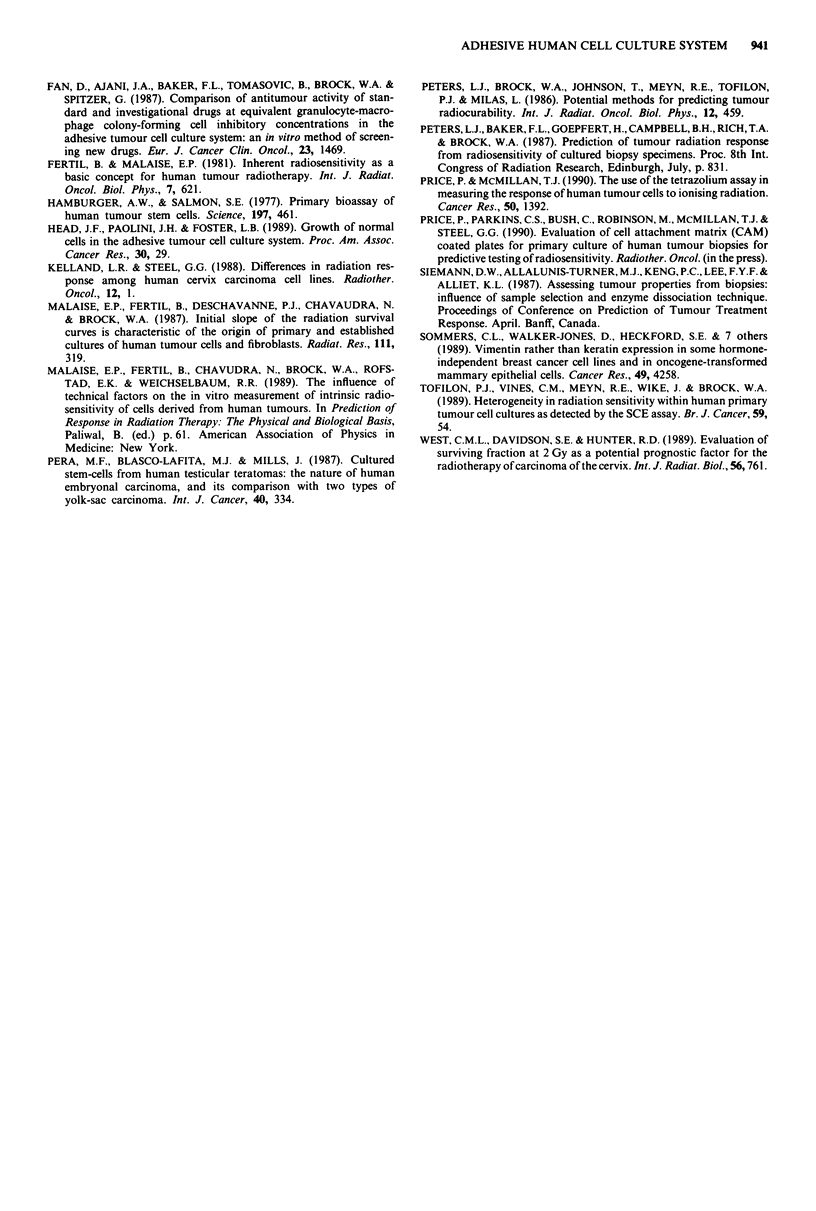# Growth and radiosensitivity testing of human tumour cells using the adhesive tumour cell culture system.

**DOI:** 10.1038/bjc.1990.413

**Published:** 1990-12

**Authors:** C. S. Parkins, G. G. Steel

**Affiliations:** Institute of Cancer Research, Sutton, Surrey, UK.

## Abstract

**Images:**


Br. J. Cancer (1990), 62, 935–941 © Macmillan Press Ltd., 1990

### Growth and radiosensitivity testing of human tumour cells using the adhesive tumour cell culture system

C.S. Parkins & G.G. Steel

*Institute of Cancer Research, Cotswold Road, Sutton, Surrey SM2 5NG, UK.*

**Summary** The radiosensitivity of human tumour cell lines and cells cultured from xenografts or biopsy specimens was measured using the adhesive tumour cell culture system (ATCCS). For cell lines the derived surviving fractions at 2 Gy were in good agreement with values obtained by clonogenic assay. However, the assay tended to overestimate survival at higher radiation doses, and thus to give a false impression of radioresistance. When cells taken from xenografts or tumour biopsies were cultured there was no evidence for selective growth of tumour cells: fibroblast-like cells commonly grew. Immunohistochemical staining against the intermediate filament, vimentin, supported the mesenchymal origin of the fibroblast-like cells. In cultures of artificial mixtures of tumour cells and fibroblasts, low proportions of fibroblasts were not excluded by the assay and consequently modified the radiation response. The majority of cultures grown from bladder carcinoma biopsy specimens appeared fibroblast-like, although in some cases clearly distinguishable colonies of tumour cells were also grown. In such tumour types the reliable measurement of radiosensitivity in cells taken from biopsies will require further development of techniques that allow the selective growth of tumour cells.

An interesting correlation has been found between the radio-responsiveness of different types of human tumour and their *in vitro* radiosensitivity (Fertil & Malaise, 1981; Deacon *et al.*, 1984). Within a single histological type the radiosensitivity of isolated cell lines also shows considerable variability and this may partly explain the range of responses observed after clinical radiation therapy. The goal of predictive testing is to quantify the *in vitro* radiosensitivity of tumour cells in a way that is predictive for the outcome after therapy. This might then lead on to the selection of an altered treatment for radio-resistant tumours.

A wide variety of assays to measure the radiosensitivity of tumour cells have been proposed (see Peters *et al.*, 1986, for review) of which the adhesive tumour cell culture system (ATCCS) developed by Baker *et al.* (1986) has been reported as a major advance (Baker *et al.*, 1985, 1988*a*; Brock *et al.*, 1985; Ajani *et al.*, 1987; Malaise *et al.*, 1987; Peters *et al.*, 1987). The growth of cells adhering to a coated or uncoated plastic surface is a standard practice in tissue culture; ATCCS will here refer to the method described by the Houston group.

The ATCCS has been developed and investigated by Baker and colleagues for both radiosensitivity and chemosensitivity testing (Baker *et al.*, 1986; Ajani *et al.*, 1987; Fan *et al.*, 1987). An improved method for growth of human tumour cells has been claimed using a coating of cell adhesive matrix (CAM) on the surface of multi-well plates and a comprehensive range of additives in the culture medium. Cells are released from biopsy samples by enzymatic digest. Over the culture period the assay conditions are reported to enhance the growth of human tumour cells.

The ATCCS is a growth assay rather than a clonogenic assay and relies on the selective growth of tumour cells. A wide spectrum of tumours can be cultured (e.g. head and neck, melanoma, colorectal tumours) and a value representing radiosensitivity can be obtained for individual patients' tumours at around 14 days after culturing.

Primary cultures of human tumour cells have been difficult to obtain because of variable growth *in vitro*. This led Courtenay and Mills (1978) to develop a soft agar cloning assay which has been subsequently confirmed as an improvement over the Hamburger and Salmon (1977) assay. In the Courtenay and Mills assay tumour cells grow as spherical colonies in an agar layer whilst the growth of cells that require anchorage to a solid substrate (e.g. fibroblasts) is inhibited. Unfortunately, clonogenic assays generally give low plating efficiencies, often in the range of 0.1% or less (Courtenay & Mills, 1978; West *et al.*, 1989). Furthermore, this assay often requires a culture period of around 4 weeks and is therefore difficult to incorporate into treatment planning.

This paper presents our experience of using the ATCCS since late 1988 when the CAM coated plates were made available to us. Our experiments were designed to investigate radiosensitivity parameters derived using the ATCCS and to compare with a clonogenic assay. For this purpose two cervix tumour lines, one bladder line and two neuroblastoma lines were assayed. To investigate the response using cells taken directly from solid tumours, we have used xenografts of bladder and cervix tumours. Preliminary data will also be presented for cultures obtained from human tumour biopsies.

### Materials and methods

Culture-adapted tumour cell lines derived from biopsies of cervix (HX156 and HX171), bladder (MGH-U1) and neuroblastoma (SKN-SH and HX142) were used in this study (Kelland & Steel, 1988; Deacon *et al.*, 1985). These cell lines were cultured (between passage numbers 5 and 150) in either Dulbecco's modified MEM or Ham's F-12 media containing 10% fetal calf serum and cultured in a gassing incubator operating at 10% carbon dioxide, ~1% oxygen at 37°C. Human fetal lung fibroblasts (HFL), in early passage, were kindly provided by Prof. J.R. Roberts at this Institute.

The ATCCS was performed according to the description of Baker *et al.* (1986). Briefly, cells were seeded into 24-well CAM-coated plates (Baker's Dozen, Houston, TX, USA) at four different dilutions in their respective media. The addition of methyl cellulose to this attachment medium is reported to aid even distribution of cells on the culture surface. However we did not find it aided the pattern of cell attachment and was therefore omitted from this study. In some cases biopsy digests were seeded onto 24-well plates that had been coated with 0.5 mg $ml^{-1}$ human fibronectin (Collaborative Research, USA).

For solid tumour digests, the culture medium was alpha-modified Eagle's MEM, porcine sera, with the addition of EGF, β-oestradiol, transferrin, hydrocortisone, insulin, Hepes as described by Baker *et al.* (1988*b*). After 1 day the culture medium was renewed and the plates were irradiated with

Correspondence: C.S. Parkins.
Received 29 May 1990; and in revised form 17 July 1990.

graded doses of 240 kVp X-rays (HVL = 2 mmCu). Each plate was held in a specially constructed jig overlaid with absorbers to obtain graded radiation doses among the culture wells. In this way each plate was given five dose levels (0, 0, 2, 3, 4, 6 Gy) at each of four different numbers of cells plated. Cultures were observed frequently during the 14 day culture period. At the end of this period cells were removed from a control (0 Gy) well and ethanol fixed for subsequent DNA analysis. The remaining wells were washed, fixed and stained with either 0.5% crystal violet or Methylene Blue. After drying, these cultures were microscopically examined and the morphology of the cells observed. These plates were sent to Dr W. Brock (at the M.D. Anderson Hospital, Houston) to measure the staining density (integrated optical density, IOD) of each well using a Magiscanner (Joyce-Loebl, Gateshead, UK). The relationship between IOD and cell innoculum size was fitted by linear regression. Small corrections were applied to the data for each plate representing the IOD for the stained plastic surface alone and for the variation in cell number initially seeded per well using $^{3}$H-TdR suicide (Baker *et al.*, 1986). Cytotoxicity was estimated from the ratio of the slope of the regression fit for the irradiated wells to that for the control wells. The derived radiation response was therefore termed 'slope ratio' (SR) and individual values fitted using a linear-quadratic least-squares regression program.

Xenografts of human bladder carcinomas (RT112, BT14) and a breast carcinoma (HX99) were grown subcutaneously in nude mice. At a tumour diameter of about 8 mm the mice were killed and the tumour dissected. Human tumour biopsies were collected and stored in culture media. If it was not possible to commence enzyme digestion on the same day as collection the samples were stored at + 4°C overnight and the digest started the next day. Solid tumour specimens were weighed and chopped, followed by digestion using 0.5% collagenase (Boehringer) and 0.004% DNAase (Sigma) for up to 6 h at 37°C. The viable cell count was made by dye exclusion under phase contrast microscopy. A sample of the digest was also prepared and ethanol-fixed for subsequent DNA content measurements.

Cells obtained from the enzyme digest were also added to Petri dishes containing glass multiwell slides (Flow). When the cultures were almost confluent, the cell layer was fixed in acetone and stored at − 20°C until histochemistry was performed. Intermediate filament staining against cytokeratin and vimentin was conducted using an indirect immunoperoxidase reaction using monoclonal antibodies against cytokeratin (Dako-CK) or vimentin (Dako-Vimen) (Pera *et al.*, 1987) (antibodies supplied by Dakopatts, Copenhagen).

Experiments were also performed using mixtures of fibroblasts and human tumour cell lines. Between 0 and 20% human fetal lung fibroblasts were added to a range of human tumour cell innocula to represent the level of 'contamination' that might be present after enzyme digest of solid tumour biopsies. These cultures were grown and stained according to the ATCCS procedure and IOD was measured for each mixture.

Radiation cell survival was also measured for six further human tumour cell lines: HX156, HX171 (cervix carcinomas), RT112, MGH-U1 (bladder carcinomas), HX142, SKN-SH (neuroblastomas) using a standard colony assay. Cervix tumour cell lines were grown in appropriate media supplemented with $2 \times 10^{5}$ per 5 ml heavily irradiated 3T3 mouse fibroblast cells (Kelland & Steel, 1988). Colonies of greater than 50 cells were scored about 14 days after irradiation.

### Results

### *Radiation response of tumour cell lines*

Implicit in the use of the ATCCS is the requirement that the staining density (IOD) at the end of the culture period is related to cell number. Figure 1 shows the correlation between IOD and cervix tumour cell number measured in duplicate wells on the day of assay. The relationship is close to linear for each of the two cell lines, although the data for HX156 indicate that IOD plateaued at a value of about 3,000 as the cultures became confluent.

**Figure 1** Relationship between integrated optical density (IOD) and cell number at the end of the culture period. ○, HX156; □, HX171.

Two examples of the use of the CAM IOD values to calculate radiation response are shown in Figure 2. The data were usually consistent with a linear relationship between the number of cells plated and IOD value. Radiation response was calculated from the ratio of the slopes of these lines. It can be seen from these two examples that the slope ratios show a curvilinear relationship with dose; the curve for HX142 is steeper than for HX156. The effect calculated at 2 Gy ($SR_2$) using linear-quadratic equation was 0.41 and 0.74 respectively.

Table I shows the correlation between the ATCCS and clonogenic assay of radiation response for two cervix and two neuroblastoma cell lines. The agreement is fairly good, suggesting that the ATCCS assay can discriminate between cell lines that differ as widely as the cervix tumours and neuroblastomas. There was a tendency for the $SR_2$ values to be slightly greater than the $SF_2$ values; this is not surprising and it is not necessary for the scaling of these parameters to be the same in order for the ATCCS assay to be useful. We conclude that on the basis of these tests the ATCCS assay performed well in evaluating the radiosensitivity of established tumour cell lines.

### *Growth of artificial mixtures of tumour cells and fibroblasts*

We have attempted to mimic the cell population obtained from a human tumour biopsy by mixing human tumour and human fibroblast cell lines. Figure 3 shows the appearance of such a culture, HX156 cervix carcinoma cells mixed with 15% HFL fibroblasts. Epithelial and stromal areas can clearly be distinguished. To test whether the ATCCS shows specificity of growth between tumour cells and fibroblasts, mixed cultures were set up containing 0, 5, 10, 15 or 20% of fibroblasts and the cultures grown for the specified period. Figure 4 shows the IOD measurements for these mixed cell cultures. The fibroblasts alone grew quickly and they reached confluence (and IOD values in excess of 3,500) within a culture period of 14 days from small numbers of cells seeded. For any seeded number of tumour cells the optical density at

**Table I** Comparison of ATCCS and clonogenic assay

| | *CFE (%)* | | *Radiation response* | |
|---|---|---|---|---|
| *Cell line* | *Nunc* | *CAM* | $SR_2$ | $SF_2$[b] |
| HX156 | 9 (11)[a] | 14 | 0.79, 0.80 | 0.73 |
| HX171 | – (44) | – | 0.72, 0.85 | 0.62 |
| HX142 | 16 | 16 | 0.31, 0.41 | 0.31 |
| SKN-SH | 42 | 14 | 0.36, 0.49 | 0.19 |

[a]Values in parenthesis represent colony forming efficiency with heavily irradiated feeder cells. [b]Typical value from clonogenic assays.

14 days increased progressively with the number of fibroblasts added. The magnitude of this effect was large: 5% of fibroblasts was equivalent to increasing the seeded tumour cell number from approximately 6,000 to 25,000.

**Figure 2** Evaluation of the ATCCS assay in two human tumour cell lines. The radiation response is measured from the reduction in slope of IOD *vs* cell number (upper panels). For HX156 (cervix carcinoma) and HX142 (neuroblastoma) the $SR_2$ were 0.80 and 0.41 respectively (lower panels). Dashed line shows linear-quadratic fit to the data.

**Figure 3** Photomicrograph of cervix tumour cell line (HX171) grown with an admixture of 15% HFL fibroblasts. The tumour cells grew in tight epithelial colonies surrounded by the more elongated fibroblasts (mag. × 192).

**Figure 4** Growth of artificial mixtures of human cervix carcinoma (HX156) cells and HFL fibroblasts. The IOD at 14 days is plotted against the number of *tumour* cells plated; ---O--- shows similar results for fibroblasts alone. The proportion of fibroblasts was: ● 0%, △ 5%, ▲10%, □ 15%, ■ 20%.

**Figure 5** Phase contrast photomicrograph of cells cultured from a bladder tumour xenograft. The tumour cells (RT112) grew in tight epithelial colonies surrounded by the more elongated mouse fibroblasts (mag. × 192).

### *Growth on CAM plates of cells taken from xenografts and biopsies*

Enzyme digests were performed on two types of breast and two types of bladder tumour xenografts and a variety of human tumour biopsy specimens; bladder (50), cervix (5), head and neck (5). Most biopsy specimens yielded very few dye-excluding (viable) cells which attached to the culture surface. This was evident by the large amount of debris removed at the media change at 1 day after seeding. Cell

**Figure 6** Evaluation by the ATCCS method of radiation response of cells cultured from a bladder tumour xenograft. Dashed line shows linear-quadratic fit to the data.

growth during the 14 days was very variable, ranging from only single cells to confluent layers of fibroblast-like cells. In general the growth from xenograft samples was best. Figure 5 shows the morphology of cells grown from a bladder tumour xenograft (RT112). Two distinct morphological cell types can be identified. The closely packed polygonal cells are colonies of tumour cells whereas the less densely packed cells are fibroblasts. Neither of the cell lines changed their morphology when grown in the presence of the other. Similarities can be seen between the cells in Figure 5 and the mixtures of HX156 and HFL cell lines shown in Figure 3. The radiation response of the RT112 xenograft cultures is shown in Figure 6 (symbols denote results from duplicate plating). The resultant radiosensitivity at 2 Gy is more resistant than that found when the tumour cell line is cultured alone ($SF_2 = 0.5$).

Figure 7 shows the morphology of cells grown from human tumour biopsies. Initial experiments using the enzyme cocktail used for the Courtenay-Mills assay (collagenase, DNAase, pronase) yielded many cultures of fibroblast-like cells. Omitting pronase from the cocktail resulted in better growth of the epithelial cells, although fibroblast-like cells were still present. A wide heterogeneity in cellular morphology was observed using this culture assay. Using biopsies of bladder and other tumour types it was common to observe colonies of densely packed small polygonal epithelial cells surrounded by other more diffuse fibroblastic cells. These cultures showed much lower growth and CFE than either cell lines or xenografts. For a small number of these cultures the radiation response was calculated at 2 Gy ($SR_2 = 0.6-0.8$) although the meaning of such a value derived from a mixture of cell types is difficult to interpret. This problem may be overcome using a screening test to determine the karyotype of the cultured cells before interpretation of the radiation response. A wider report of the evaluation of CAM plates from our group is in preparation (Price *et al.*, 1990).

### *Identification of cells growing on CAM plates*

Immunocytochemical staining for intermediate filaments was used to distinguish between cells of epithelial or mesenchymal origin. Epithelial cells should be antibody positive for cytokeratin and negative for vimentin but mesenchymal cells should show the opposite staining pattern. Figure 8 shows the antibody staining pattern for tumour and fibroblast cell lines and primary cultures obtained from biopsy specimens. The cervix tumour cell line HX156 has a highly positive reaction for cytokeratin and negative for vimentin whereas the reverse is true for the fibroblast line. When the xenograft

**Figure 7** Phase contrast photomicrograph of cells cultured using the ATCCS from biopsy specimens of bladder, larynx and nephroblastoma tumours (mag. × 160). Tumour cell colonies grew in tight epithelial colonies surrounded by the more elongated fibroblasts. **a**, bladder culture (case 7789); **b**, larynx culture (case 3889) (mag. × 252); **c**, bladder culture (case 12789); **d**, nephroblastoma culture (case 291189).

culture was stained using these two antibodies it was seen that the colonies of small tightly packed cells were positive for cytokeratin and negative for vimentin. The mouse fibroblast-like cells were, as expected, positive for vimentin (using anti-mouse antibody) and negative for cytokeratin.

It was not possible to apply the immunohistochemical staining directly to the cells adherent to the CAM coated surface due to the action of the acetone fixative on the plastic surface. However, primary cultures were also grown on glass multiwell slides and the colonies that grew were mainly

**Figure 8** Immunohistochemical staining against epithelial (cytokeratin) and mesenchymal (vimentin) intermediate filaments. **a**, Cervix tumour cell line (HX156) stained against cytokeratin. **b**, Human fetal lung fibroblasts stained against vimentin. **c**, Tumour cells from primary culture of nephroblastoma biopsy stained against cytokeratin. **d**, Fibroblast cells from primary culture of bladder biopsy stained against vimentin.

fibroblastic, staining positive for vimentin and negative for cytokeratin. In those cases where mixed tumour and fibroblast morphologies appeared on the CAM plate, similar morphologies appeared on the glass slides. Cells of tumour morphology were positive for cytokeratin and those of fibroblastic morphology, positive for vimentin. No cross-reactivity of the antibodies was detected.

### *DNA index measurements*

Xenografts of RT112 were analysed according to the ATCCS yielding both diploid and aneuploid cells as measured by flow-cytometry (DNA index 1.5). However, no significant increase in the proportion of aneuploid cells was found at the end of the 14 day ATCCS culture period.

### Discussion

The ATCCS is reported to offer two major advantages over other assays: (1) its ability to grow and quantify human tumour cells from a wide range of biopsy material and (2) inhibition of growth of cell types other than tumour cells. In order to provide meaningful data this growth assay should reflect the underlying clonogenic response of the tumour cell population. Growth assays only yield accurate data when the assay conditions, e.g. the initial cell inoculum, cell growth rate, culture period and method of assessing cell number are optimal for the cells being assessed (Price & McMillan, 1990). An overestimation of radioresistance would be observed if the cell number is measured at a time when growth in the control or irradiated wells is not exponential or if the staining density is affected by doomed or dead cells. The choice of a fixed assay time of 14 days would be inappropriate for cells with a range of doubling times and this effectively limits the range of surviving fraction that can be measured. The choice of assay time also depends upon the initial number of cells seeded. Using human tumour cell lines we found the derived dose-response to be in close agreement with that from a clonogenic assay although the sensitivity at higher doses was underestimated. The relationship between IOD and cell inoculum size may be poorly fitted using linear regression due to confluency. Indeed this is probably the explanation for the observed radioresistance at high doses for the cell lines in this study, in agreement with Malaise *et al.* (1989). The derived radiosensitivity parameter would be more accurately termed the IOD slope ratio. The response at 2 Gy has therefore been termed $SR_2$ and not $SF_2$ within this study. For cells of unknown growth or cloning efficiency (CFE) misleading values of radiosensitivity may occur and it would be difficult to estimate the potential effect of the aforementioned variables.

For our cell lines no significant increase in CFE was observed using the CAM coating compared to growth on tissue culture grade surfaces. However, when cells of mixed origin, e.g. epithelial and mesenchymal were seeded, the proportion of fibroblast cells attached to the CAM surface increased accordingly. The fibroblast line investigated (HFL), although untransformed, are culture adapted and it may be argued that their growth is not representative of the fibroblasts obtained from primary culture. However, the frequent observation of fibroblasts from both biopsy samples and xenografts suggests that the assay does not inhibit their growth and therefore the use of fibroblast cell lines is justified in evaluating the assay. Mouse fibroblasts are normally considered to be difficult to grow in culture and their appearance, and positive identification using an antibody against mouse vimentin, in cultures from xenografts indicates the lack of success of the assay. Primary culture growth was also observed using alternatives to CAM surface using a range of surface coatings, e.g. collagen, fibronectin or vitronectin (obtained from the growth media of a germ cell tumour line isolated by Dr M. Pera at this Institute). These coatings increased the attachment of cells compared with bare plastic but none showed a specific affinity for tumour cells which was not also shown for fibroblasts.

Our preliminary results of cell growth from human tumour biopsies have shown great heterogeneity in morphology and growth rate. In some cases only fibroblast-like cells grew and in others colonies of epithelial, polygonal, tumour cells grew. Explanations for our observations of mixed cell morphology compared to that reported by the group at Houston are: (1) that our culture conditions are different; (2) that the tumour type we digest contains a high proportion of fibroblasts; or (3) that mixed cell morphology exists in cultures from both groups.

Technical differences between the two studies were few. The low oxygen tension used in this study is equal to that used in the Courtenay-Mills assay in which it was found to promote the growth of human tumour cells. Since there is evidence that growth in atmospheric oxygen is equally good (Besch *et al.*, 1986) it is not expected that this would be an important factor. Also differences in the batch of porcine sera are unlikely to suppress fibroblast growth in USA but promote their growth in UK. There were no differences in the enzyme cocktail used although it is reported that the yield of cell types after enzymatic digest of solid tumours is variable and dependent upon the cocktail of enzymes applied (Siemann *et al.*, 1987).

The second factor may be the most important difference between the two institutes, i.e. that the majority of biopsy samples we have attempted to culture are from early stage I or II bladder carcinoma. The undifferentiated nature of these tumours and the mode of biopsy (transurethral resection using cautery wire) may explain the high incidence of fibroblast growth from the bladder biopsy specimens. Better growth of epithelial tumour cells was obtained from the small number of head and neck and cervix tumours, although fibroblasts were still evident. Mixed cell morphology has been reported for cells grown from lung and breast tumours using the ATCCS assay (Head *et al.*, 1989). The explanation that mixed cultures grew in both groups needs further investigation.

A recent report from Tofilon *et al.* (1989) investigated the production of SCE in primary cultures obtained using the ATCCS. A wide heterogeneity of SCE induction was observed and this was explained as indicating heterogeneity of radiosensitivity from the primary tumour culture. Unless the proportion of any contaminating cell type, e.g. fibroblasts, was assessed the findings cannot exclude the alternative conclusion that the cultures contained a variable proportion of diploid fibroblasts and that these were responsible for the heterogeneity in SCE induction.

Reduction in fibroblast cell contamination in primary culture may be expected if a screening test were applied to detect and discard non-tumour cells before they were seeded in the ATCCS. However our attempts to separate mixtures of tumour cell lines from added fibroblasts, using Percoll density centrifugation, were not successful. Until there is evidence that the radiosensitivity of tumour cells and fibroblasts derived from the same patient are related in some way the validity of the calculated $SR_2$ value from a mixed population is uncertain. Morphological appearance alone is insufficient to determine the proportion of cell types obtained in a primary culture. We have not observed any change in the growth of human tumour and fibroblast cell lines on CAM plates and therefore believe that cells with elongated cytoplasmic processes (that typically grow in swirls parallel to each other) are truly fibroblasts.

To confirm this conclusion we investigated the use of staining against intermediate filaments as a differential marker of tumour cells and fibroblasts. Fibroblast cells express the intermediate filament vimentin and epithelial tumour cells express cytokeratin. We have not found any cross reaction between the antibodies and none of our epithelial tumour cell lines were positive for vimentin but all were positive for cytokeratin. This discrimination is in contrast to the report by Sommers *et al.* (1989), who reported vimentin rather than cytokeratin expression in a small proportion of hormone independent and oncogene-transformed cell lines. Since the plastic plate supporting the CAM surface is not impervious to the fixative necessary for intermediate filament staining we have not been able to perform the definitive test of staining against intermediate filaments directly on primary cultures grown on CAM plates. However, the human tumour, fibroblast and xenograft cells grown showed unchanged staining patterns when grown on glass surfaces. Glass microscope cover slips coated with CAM are currently being evaluated at the M.D. Anderson Hospital, Houston to address this question. Our attempts to transfer the CAM from the supplied plates and re-coat glass cover slips were not successful.

In conclusion we have found that: (1) the radiosensitivity ($SR_2$) of human tumour cell lines is in close agreement with their response using a clonogenic assay; (2) the ATCCS was unable to selectively grow human tumour cell lines when the fibroblast contamination was 5% or greater; (3) measurement of cell number using staining density is misleading if confluency is reached or if both tumour cells and fibroblasts are grown; (4) a high incidence of fibroblasts was observed when human tumour xenografts were assayed; and (5) both tumour and fibroblast cells were grown from human tumour biopsies. Our success in growing tumour cells from human tumour biopsies is much poorer than that reported by Houston. We feel that this cannot totally be explained by the slight differences in culture conditions or in the tumour types chosen in our preliminary study.

This work was funded by NCI grant no. Ro1CA26059. The assistance of Dr W.A. Brock, and colleagues, at the M.D. Anderson Cancer Centre, Houston, in measuring the staining density of the cultures is gratefully acknowledged.

### References

AJANI, J.A., BAKER, F.L., SPITZER, G. & 6 others. (1987). Comparison between clinical response and in vitro drug sensitivity of primary human tumours in the adhesive tumour cell culture system. *J. Clin. Oncol.*, **5**, 1912.

BAKER, F., SPITZER, G., AJANI, J. & 5 others (1985). Adhesive tumour cell culture system: successful primary culture of human tumour cells on an adhesive substrate. *Proc. Am. Assoc. Cancer Res.*, **26**, 367.

BAKER, F.L., SPITZER, G., AJANI, J.A. & 8 others (1986). Drug and radiation sensitivity measurements of successful primary monolayer culturing of human tumour cells using cell-adhesive matrix and supplemented medium. *Cancer Res.*, **46**, 1263.

BAKER, F.L., SPITZER, G., AJANI, J.A. & BROCK, W.A. (1988*a*). Drug and radiation sensitivity testing of primary human tumour cells using the adhesive tumour cell culture system (ATCCS). In *Prediction of Response to Cancer Therapy*, p. 105. Alan R. Liss: New York.

BAKER, F.L., AJANI, J., SPITZER, G. & 4 others (1988*b*). High colony-forming efficiency of primary human tumour cells cultured in the adhesive-tumour-cell culture system: improvements with medium and serum alterations. *Int. J. Cell. Cloning*, **6**, 95.

BESCH, G.J., TANNER, M.A., HOWARD, S.P., WOLBERG, W.H. & GOULD, M.N. (1986). Systematic optimisation of the clonal growth of human primary breast carcinoma cells. *Cancer Res.*, **46**, 2306.

BROCK, W.A., BHADKAMKAR, V.A., WILLIAMS, M., SPITZER, G. & BAKER, F. (1985). Radiosensitivity testing of primary cultures derived from human tumours. In *Progress in Radio-Oncology, vol. 3*, Karcher, K.H. (ed.). ICRO: Vienna.

COURTENAY, V.D. & MILLS, J. (1978). An *in vitro* colony assay for human tumours grown in immune suppressed mice and treated *in vivo* with cytotoxic agents. *Br. J. Cancer*, **37**, 261.

DEACON, J., PECKHAM, M.J. & STEEL, G.G. (1984). The radioresponsiveness of human tumours and the initial slope of the cell survival curve. *Radiother. Oncol.*, **2**, 317.

DEACON, J.M., WILSON, P.A. & PECKHAM, M.J. (1985). The radiobiology of human neuroblastoma. *Radiother. Oncol.*, **3**, 201.

FAN, D., AJANI, J.A., BAKER, F.L., TOMASOVIC, B., BROCK, W.A. & SPITZER, G. (1987). Comparison of antitumour activity of standard and investigational drugs at equivalent granulocyte-macrophage colony-forming cell inhibitory concentrations in the adhesive tumour cell culture system: an *in vitro* method of screening new drugs. *Eur. J. Cancer Clin. Oncol.*, **23**, 1469.

FERTIL, B. & MALAISE, E.P. (1981). Inherent radiosensitivity as a basic concept for human tumour radiotherapy. *Int. J. Radiat. Oncol. Biol. Phys.*, **7**, 621.

HAMBURGER, A.W., & SALMON, S.E. (1977). Primary bioassay of human tumour stem cells. *Science*, **197**, 461.

HEAD, J.F., PAOLINI, J.H. & FOSTER, L.B. (1989). Growth of normal cells in the adhesive tumour cell culture system. *Proc. Am. Assoc. Cancer Res.*, **30**, 29.

KELLAND, L.R. & STEEL, G.G. (1988). Differences in radiation response among human cervix carcinoma cell lines. *Radiother. Oncol.*, **12**, 1.

MALAISE, E.P., FERTIL, B., DESCHAVANNE, P.J., CHAVAUDRA, N. & BROCK, W.A. (1987). Initial slope of the radiation survival curves is characteristic of the origin of primary and established cultures of human tumour cells and fibroblasts. *Radiat. Res.*, **111**, 319.

MALAISE, E.P., FERTIL, B., CHAVUDRA, N., BROCK, W.A., ROFSTAD, E.K. & WEICHSELBAUM, R.R. (1989). The influence of technical factors on the in vitro measurement of intrinsic radiosensitivity of cells derived from human tumours. In *Prediction of Response in Radiation Therapy: The Physical and Biological Basis*, Paliwal, B. (ed.) p. 61. American Association of Physics in Medicine: New York.

PERA, M.F., BLASCO-LAFITA, M.J. & MILLS, J. (1987). Cultured stem-cells from human testicular teratomas: the nature of human embryonal carcinoma, and its comparison with two types of yolk-sac carcinoma. *Int. J. Cancer*, **40**, 334.

PETERS, L.J., BROCK, W.A., JOHNSON, T., MEYN, R.E., TOFILON, P.J. & MILAS, L. (1986). Potential methods for predicting tumour radiocurability. *Int. J. Radiat. Oncol. Biol. Phys.*, **12**, 459.

PETERS, L.J., BAKER, F.L., GOEPFERT, H., CAMPBELL, B.H., RICH, T.A. & BROCK, W.A. (1987). Prediction of tumour radiation response from radiosensitivity of cultured biopsy specimens. Proc. 8th Int. Congress of Radiation Research, Edinburgh, July, p. 831.

PRICE, P. & MCMILLAN, T.J. (1990). The use of the tetrazolium assay in measuring the response of human tumour cells to ionising radiation. *Cancer Res.*, **50**, 1392.

PRICE, P., PARKINS, C.S., BUSH, C., ROBINSON, M., MCMILLAN, T.J. & STEEL, G.G. (1990). Evaluation of cell attachment matrix (CAM) coated plates for primary culture of human tumour biopsies for predictive testing of radiosensitivity. *Radiother. Oncol.* (in the press).

SIEMANN, D.W., ALLALUNIS-TURNER, M.J., KENG, P.C., LEE, F.Y.F. & ALLIET, K.L. (1987). Assessing tumour properties from biopsies: influence of sample selection and enzyme dissociation technique. Proceedings of Conference on Prediction of Tumour Treatment Response. April. Banff, Canada.

SOMMERS, C.L., WALKER-JONES, D., HECKFORD, S.E. & 7 others (1989). Vimentin rather than keratin expression in some hormone-independent breast cancer cell lines and in oncogene-transformed mammary epithelial cells. *Cancer Res.*, **49**, 4258.

TOFILON, P.J., VINES, C.M., MEYN, R.E., WIKE, J. & BROCK, W.A. (1989). Heterogeneity in radiation sensitivity within human primary tumour cell cultures as detected by the SCE assay. *Br. J. Cancer*, **59**, 54.

WEST, C.M.L., DAVIDSON, S.E. & HUNTER, R.D. (1989). Evaluation of surviving fraction at 2 Gy as a potential prognostic factor for the radiotherapy of carcinoma of the cervix. *Int. J. Radiat. Biol.*, **56**, 761.